# Understanding of cell death induced by the constituents of *Taxus yunnanensis* wood

**DOI:** 10.1038/s41598-022-09655-2

**Published:** 2022-04-15

**Authors:** Yukihiro Akao, Riyako Terazawa, Nobuhiko Sugito, Kazuki Heishima, Kohei Morikawa, Yuko Ito, Ryoko Narui, Reo Hamaguchi, Takahiro Nobukawa

**Affiliations:** 1 United Graduate School of Drug Discovery and Medical Information Sciences, Gifu University, 1-1 Yanagido, Gifu, Japan; 2 Department of General and Gastroenterological Surgery, Osaka Medical and Pharmaceutical University, Takatsuki, Japan; 3 Karasuma Wada Clinic, Nakagyo-ku, Kyoto, 604-0845 Japan; 4 Kotosugi Co., Ltd, Sagamihara, Japan

**Keywords:** Cancer, Cell biology, Drug discovery, Molecular biology

## Abstract

The ethanol extract from the wood of *Taxus Yunnanensis* (TY) induced apoptosis in all cancer cell lines tested, which was mainly due to activation of an extrinsic pathway in human colon cancer DLD-1 cells. The extrinsic pathway was activated by the upregulation of the expression levels of Fas and TRAIL/DR5, which led to the activation of caspase-8. Of note, the machinery of this increase in expression was promoted by the upregulation of MIR32a expression, which silenced MIR34a-targeting E2F3 transcription factor. Furthermore, ectopic expression of MIR32a or siR-E2F3 silencing *E2F3* increased Fas and TRAIL/DR5 expression. Thus, the extract activated the extrinsic pathway through the MIR34a/E2F3 axis, resulting in the autocrine and paracrine release of TRAIL, and upregulated expression of death receptors Fas and DR5 in the treated DLD-1 cells, which were functionally validated by Fas immunocytochemistry, and using anti-Fas and anti-TRAIL antibodies, respectively. In vivo, TY showed significant anti-tumor effects on xenografted and syngeneic model mice. The extract may also aid in chemoprevention by selectively making marked tumor cells susceptible to the tumor immunosurveillance system.

## Introduction

The extract of *Taxus yunnanensis* wood has been used in traditional Chinese medicine by people in Yunnan Province for treatment of the kidneys, diabetic ailments and other diseases. Currently, around 23,000 people in Japan are taking it for healthcare. Chemical studies of the constituents and their functions, such as anti-rheumatic effects, have been reported^1–6^. On the other hand, paclitaxel is a potent anticancer drug that was initially isolated from the bark of *Taxus brevifolia*^1^. Since the discovery of paclitaxel in 1971^1^, much effort has been devoted to isolate new taxane diterpenes with similar anticancer activity. As a result, more than 350 taxane diterpenoids have been found from the bark of Taxus plants^2–4^. The activity of most diterpenoids from bark is markedly high and toxic to healthy cells^5^. However, the wood extract exerted minor effects on normal cells, but it induced apoptosis mostly in cancer cells^5^. This difference in anti-proliferative activity is due to differences in the constituents between the bark and wood. Although the pathways of cell growth and apoptosis have been extensively investigated, it is still unclear how growth inhibition and apoptosis are regulated. Cancer cells enter the cell cycle and progress by being stimulated by certain growth factors. On the other hand, apoptosis can be initiated through one of two pathways: intrinsic pathway and extrinsic pathway. The intrinsic signaling pathways that initiate apoptosis through non-receptor-mediated stimuli that produce intracellular signals and are mitochondrial-initiated events. Extrinsic pathways involve two pathways, death-receptor or non-death-receptor mediated signal. In death-receptor mediated apoptosis of extrinsic pathway, two types of the pathways have been defined: to be independent of mitochondria, called Type I and mitochondria dependent, called Type II. In the present study, we demonstrated that the apoptosis induced by the wood extract is mainly through an extrinsic pathway induced by the greater upregulation of MIR34a silencing *E2F2* (MIR34a/E2F axis). It has been reported that phytochemicals affect epigenetic modifications, such as DNA methylation, and histone modifications in addition to the regulation of the expression of non-coding microRNAs (miRNAs) for the prevention of cancer^6–8^. MiRNAs control numerous biological processes such as cell proliferation, apoptosis, and cell differentiation. Due to their significant roles in cell physiology, alterations in expression levels are directly related to cancer progression. Many phytochemicals have promise in regulating DNA methylation and histone modification in carcinogenesis^6–8^, suggesting the use of dietary-based phytochemicals as potent and effective chemopreventive medicines. In this study, the constituents of the extract upregulated MIR34a, which silenced the transcription factor E2F3. In addition, death factors/receptors, such as FasL/Fas and TRAIL/DR5, were negatively regulated by E2F3 after treatment in human colon cancer DLD-1 cells. Consequently, the extract induced apoptosis via the activation of caspase-8 only in cancer cells through an extrinsic pathway by increasing the expression of such death factors/receptors. This machinery is of great interest from the viewpoint of tumor immunology and cancer prevention because of the minor effects on healthy cells.

## Materials and methods

### Preparation for extract of *Taxus yunnanensis*

The active ingredients of *Taxus yunnanensis* were extracted from dried woodchips in 60% ethanol for 2 days. After evaporating the solvent, the bulk extract was weighed and diluted in DMSO. The extract (TY) was stored at − 30 °C until use.

### Cell lines and culture

DLD-1, DU145, K562, RPMI8226, and the other cells used in this study were cultured in RPMI1640 (Wako, Osaka, Japan) supplemented with 10% FBS (Sigma-Aldrich, MO, USA). H9c2 cells were cultured in DMEM (Wako) supplemented with 10% FBS (Sigma-Aldrich). ASF 4-1 cells were cultured in EMEM (Wako) containing 10% FBS (Sigma-Aldrich). All cell lines used were obtained from the Japanese Cancer Research Resources Bank except for DU145, which was obtained from the Riken Bioresource Research Center. Cell lines used were confirmed as being negative for mycoplasma tMycoAlert, Lonza, Basel, Switzerland). All cells were cultured in a humidified incubator with 5% CO_2_ at 37 °C.

### Analysis of TY-induced cell death

For cytotoxicity analysis, cells were seeded on a 6-well plate at a concentration of 0.5 × 10^5^ cells/mL. The cells were treated with TY (0, 1, 3, or 5 µg/mL). After a 48- or 72-h incubation, viable cells were counted using the trypan blue exclusion test. For morphological apoptotic analysis, the TY-treated and DMSO-treated cells were stained for 30 min with 5 μg/mL of Hoechst 33342 (Sigma-Aldrich) at 37 °C. After washing with PBS, nuclear morphology was imaged using a fluorescence microscope (Olympus, Shinjuku, Tokyo, Japan). The apoptotic cell percentage was calculated by counting cells with fragmented nuclei among 500 cells. For functional inhibitory assessment of apoptosis, chemical inhibitors of apoptosis (Z-VAD-FMK, Cat. 4800-520, MBL, Aichi, Japan; Z-IETD-FMK, Cat. 4805-510, MBL; Z-LEHD-FMK, Cat. 4810-510, MBL) were used. For apoptotic analysis, DLD-1 cells were seeded at a concentration of 0.5 × 10^5^ cells/mL and incubated for 3 h. Subsequently, the cells were treated with certain concentrations of Z-VAD-FMK, Z-IETD-FMK or Z-LEHD-FMK overnight. The cells were then treated with TY at 5 µg/mL for 72 h, followed by viable cell count using the trypan blue exclusion test. For further functional inhibitory assessment of apoptosis, anti-Fas activating antibody (CH11: Sigma-Aldrich, Merck)^9,10^was used for synergistic activation of extrinsic apoptosis signaling by co-treatment with TY. Anti-TRAIL blocking antibody (2E5: Enzo Life Science, Cosmo Bio, Japan)^11^ was used to inhibit extrinsic apoptosis signaling by co-treatment with TY.

### Cell-cycle analysis

Cell-cycle progression was analyzed by quantification of cellular DNA content using the propidium iodide (PI) staining method (Tali Cell Cycle Kit, Thermo Fisher Scientific, MA, USA). Briefly, DLD-1 cells treated with TY (5 or 10 µg/mL) or DMSO were washed twice with PBS and centrifuged at 500 × g for 5 min. These cells were fixed with ice-cold 70% ethanol. After overnight incubation at − 20 °C, the cells were centrifuged at 1000×*g* for 5 min at 4 °C. After a wash with 1 mL of PBS, the cells were stained with 200 μL of the Tali Cell Cycle Solution containing PI for 30 min at room temperature in the dark. Subsequently, 25 μL of stained cells was applied onto Tali Cellular Analysis Slides and analyzed with the Tali Image-Based Cytometer (Thermo Fisher Scientific), and 20 fields per sample were captured with the function of the Tali Cell Cycle Assay. The data obtained were analyzed in GraphPad Prism 8 (version 8.4.0, GraphPad Software, CA, USA).

### Immunoblot

To prepare cell lysate, DLD-1 cells treated with TY at 5 µg/mL for 24, 48, and 72 h were lysed in ice-cold RIPA buffer. In the case of tumor samples, the tumors were sonicated in 1% SDS buffer (Wako) and centrifuged at 13,000×*g* for 20 min. The protein concentrations of the supernatants were measured using the DC Protein Assay Kit (Bio-Rad, CA, USA). Protein samples (1–5 µg/lane) were subjected to sodium dodecylsulphate-polyacrylamide gel electrophoresis (SDS-PAGE). The proteins in the gel were transferred to 0.45-µm polyvinylidene fluoride membranes (Immobilon-P Membrane, EMD Millipore, MA, USA)^12^. The membranes were blocked with PVDF Blocking Reagent for Can Get Signal (TOYOBO, Osaka, Japan) for 1 h. Subsequently, the membranes were incubated overnight at 4 °C with primary antibodies at a 1:1000 dilution. The membranes were then washed 3 times with PBS containing 0.1% Tween 20, incubated further with HRP-conjugated sheep anti-mouse or donkey anti-rabbit IgG antibody (Cell Signaling Technology [CST], Inc., Danvers, MA, USA) at room temperature, and then washed 3 times with PBS containing 0.1% Tween 20. The immunoblots were visualized using the Luminata Forte Western HRP substrate (EMD Millipore)^12^. Densitometry of the immunoblots was performed using Image J (version 2.0.0-rc-69/1.52p, NIH). Primary antibodies used were as follows: anti-FAS (#8023), DR5 (#8074), TRAIL (#3219), cyclin D1 (#55506), cleaved caspase-8 (#9496), PARP (#9542), FADD (#2782), and phosphate-FADD (#2781; CST); anti-FAS ligand (#JM-3330-100) and caspase-8 (#M032-3; MEDICAL & BIOLOGICAL LABORATORIES CO., LTD., Tokyo, JP); anti-E2F3 (SC-56665; Santa Cruz Biotechnology, Santa Cruz, CA, USA); and anti-SIRT1 and anti-Ras antibodies (ab32441; Abcam, Cambridge, UK). The growth-related anti-phospho-ERK1/2 (#4370), anti-ERK1/2 (#4695), anti-phospho-AKT (Ser473) (#4060), and anti-AKT (#9272) antibodies were obtained from CST. The loading protein amount was assessed with an anti-β-actin mouse monoclonal antibody (clone AC-74, Cat. #A5316, Sigma-Aldrich). Anti-rabbit IgG (#7074) and anti-rabbit IgG, HRP-linked antibody (#7076; CST) were used as secondary antibodies.

### qRT-PCR

Total RNA was extracted from DLD-1 cells treated for 24 or 48 h with TY at 5 µg/mL. In brief, total RNA was purified using NucleoSpin miRNA (MACHEREY–NAGEL, Düren, Deutschland). The RNA concentration and integrity were assessed by UV spectrophotometry and Agilent 2100 Bioanalyzer (Agilent, CA, USA), respectively.

To detect mRNA, cDNA was reverse transcribed from 0.5 µg of the total RNA using the PrimeScript RT Reagent Kit (Takara, Shiga, Japan)^12^. The cDNA was amplified with specific primers for *FAS* (Forward, 5'-GGACCCTCCTACCTCTGGTT-3'; Reverse, 5'-ACCTGGAGGACAGGGCTTAT-3'), *TRAIL* (Forward, 5'-ATCATGGCTATGATGGAGGT-3'; Reverse, 5'-CTGTTCATACTCTCTTCGTC-3') and *ACTB* (Forward, 5'- GATTCCTATGTGGGCGACGA-3'; Reverse, 5'- AGGTCTCAAACATGATCTGGGT-3') by using Universal SYBR Select Master Mix (Applied Biosystems, Thermo Fisher Scientific, MA, USA). The signals were recorded using the TaKaRa Thermal Cycler Dice Real Time System II (Takara). Relative expression levels were quantified by the ΔCt method and normalized to *ACTB*. To detect miRNA, 25 ng of total RNA was reverse transcribed to cDNA using the TaqMan MicroRNA Reverse Transcription Kit (Applied Biosystems)^12^. Expression levels of MIR34a were assessed by performing TaqMan MicroRNA Assays (Applied Biosystems). The expression levels of MIR34a were normalized using the ΔCt method and internal control (*RNU6B*). Each measurement was performed in triplicate.

### Transfection of DLD-1 cells with miRNA or siRNA

DLD-1 cells were seeded in 6-well plates at a concentration of 0.5 × 10^5^ to 1.0 × 10^5^/1 mL/well on the day before transfection. MIR34a mimic (Applied Biosystems) or/and the inhibitor of MIR34a (anti-miR-34a, 40–80 nM; Applied Biosystems) or siRNAs for *E2F3* (siR-E2F3: 5–10 nM) were used for the transfection of the cells, which was achieved by cationic liposomes, i.e., Lipofectamine RNAiMAX (Invitrogen, Carlsberg) according to the manufacturer's lipofection protocol. The sequence of anti-miR-34a was 5′-UGGCAGUGUCUUAGCUGGUUGU-3′; and that of siRNA for *E2F3*, 5′-UAACCUUUGAUUCUCUGAAUCCUCG-3′ (siR-*E2F3*). We used nonspecific control Duplex VII (57% GC Content; Dharmacon Research, Inc., Lafayette, CO, USA) as a control. The effects of the introduction of the miRNA, siRNA, or anti-MIR34a inhibitor into the cells were assayed at 48 or 72 h after transfection.

### Assay for luciferase activity

We constructed sensor vectors by joining the region with or without a possible binding site from the 3′-UTR of human *E2F3* (No. 4271-4650) with a luciferase reporter pMIR-control vector (Ambion, Foster City, CA, USA) to examine the target sequence of MIR34a. To generate sensor vectors with four mutations in the binding site of the 3′-UTR of human *E2F3* (No. 4440-4462) for MIR34a, we mutated seed regions from CACTGCCA to CAAGTGCA (mt-*E2F3*, PrimeSTAR Mutagenesis Basal Kit; TaKaRa). The sensor vector with these mutations was submitted to Life Science Research Center, Gifu University, for DNA sequencing. The cells were seeded in 12-well plates at a concentration of 0.5 × 10^5^/well the day before transfection. The sensor vector (concentration, 0.5 μg/well) and 40 nM MIR34a or nonspecific control miRNA (Dharmacon) was used for the co-transfection of the cells with Lipofectamine RNAiMAX (Invitrogen). The measurement of the activities was described previously^12^.

### Immunocytochemistry

After DLD-1 cells were treated with TY (4, 5, or 6 µg/mL) or DMSO for 48 h, the cells were fixed with 4% formaldehyde for 15 min and then incubated in 1% BSA/10% normal goat serum/0.3 M glycine in 0.1% PBS-Tween for 1 h to permeabilize the cells and block non-specific protein–protein interactions. The cells were then incubated overnight at 4 °C with Alexa Fluor 647 Anti-Fas antibody (ab204671, Abcam) at a 1:50 dilution. Nuclear DNA was labelled with Hoechst 33342. Images were taken by confocal microscopy (Leica-Microsystems).

### Transmission electron microscopy (TEM)

After DLD-1 cells were treated with TY (5 µg/mL) or DMSO for 72 h, they were washed with ice-cold PBS. The cells were then fixed with 2% paraformaldehyde and 2.5% glutaraldehyde in 0.2 M PBS for 2 h. After washing with PBS and post-fixing in 2% osmium tetraoxide for 2 h, the cells were dehydrated with a 10% graded series of 30–100% ethanol and cleared with QY-1 (Nissin EM, Tokyo, Japan). The cells were then embedded in Epon 812 resin (TAAB Laboratories Equipment, Reading, UK) and sectioned at 70-nm thickness. The sections were stained with uranyl acetate and lead citrate. Transmission electron microscopic images were obtained with a Hitachi-7650 operating at 80 kV (Hitachi, Tokyo, Japan).

### Animal experiments

All mouse experiments were approved by the Institutional Animal Care and Use Committee of Gifu University and performed according to the NIH *Guide for the Care and Use of Laboratory Animals* (National Academies Press, 2011). Our in vivo study was reported accordance with ARRIVE guidelines (https://arriveguidelines.org).

Their care was in accordance with guidelines of Gifu University. All mice were maintained with ad libitum food and water under the standard light–dark cycle. Tumor volume was measured using the following formula: V = π/6(L × W2), where L, length; W, width of the tumor.

For the heterotopic colon cancer xenograft mice model, DLD-1 cells (5 × 10^6^ cells/100 μL of PBS) were subcutaneously injected into the right flank of BALB/c *nu/nu* mice (female, 4 weeks old, SLC; n = 5). After the mean tumor volume reached approximately 100 mm^3^, the mice were randomly separated into 2 groups. Subsequently, the mice were intraperitoneally administered (once in 2 days for a total of 3 injections) TY (50 mg/kg) or vehicle (0.1% DMSO in PBS). Two days after the final administration, the mice were sacrificed for the assessment of tumor tissues. For the heterotopic syngeneic colon cancer mice model, colon 26 cells (2 × 10^6^ cells in 100 μL of PBS) were subcutaneously inoculated into female BALB/c mice (6 weeks old, SLC, Japan). After the tumor volumes reached an average of 80 mm^3^ in size, the mice were randomly allocated to different groups based on their tumor size. The mice were treated with TY at 50 mg/kg or DMSO following an intraperitoneal daily administration protocol for 20 days. The mice were euthanized with an overdose of isoflurane (20 mL in an animal chamber; Pfizer, NY, USA). GraphPad Prism 8 (version 8.4.0, GraphPad Software, CA, USA) and JMP (version 12.2, SAS Institute, NC, USA) were used for statistical analysis.

### TUNEL staining

TUNEL staining was performed with a TUNEL assay kit (ab206386, abcam). Briefly, tissues for the TUNEL stain were fixed in 4% neutral buffered paraformaldehyde, embedded in paraffin, and sectioned at 5 μm. The slides were deparaffinized in lemosol and rehydrated by passage through graded alcohols. For antigen retrieval, the sections were incubated with Proteinase K solution. The sections were then incubated in 0.3% hydrogen peroxide in methanol for 20 min to inhibit endogenous peroxidase activity. Subsequently, the samples were labeled with TdT enzyme and FITC. After 3 washes with TBS-T, the sections were blocked with normal serum and incubated with anti-FITC antibody conjugated with HRP. After 3 washes with TBS-T, the signals were visualized with DAB chromogen solution. The slides were counterstained with hematoxylin. Histopathological images were acquired using a microscope (BZ-X700, KEYENCE).

### Statistics

Differences were statistically evaluated by one-way analysis of variance followed by the *t* test. A *P*-value of less than 0.05 was considered significant.

## Results

### TY induced cell death

**Figure 1:**
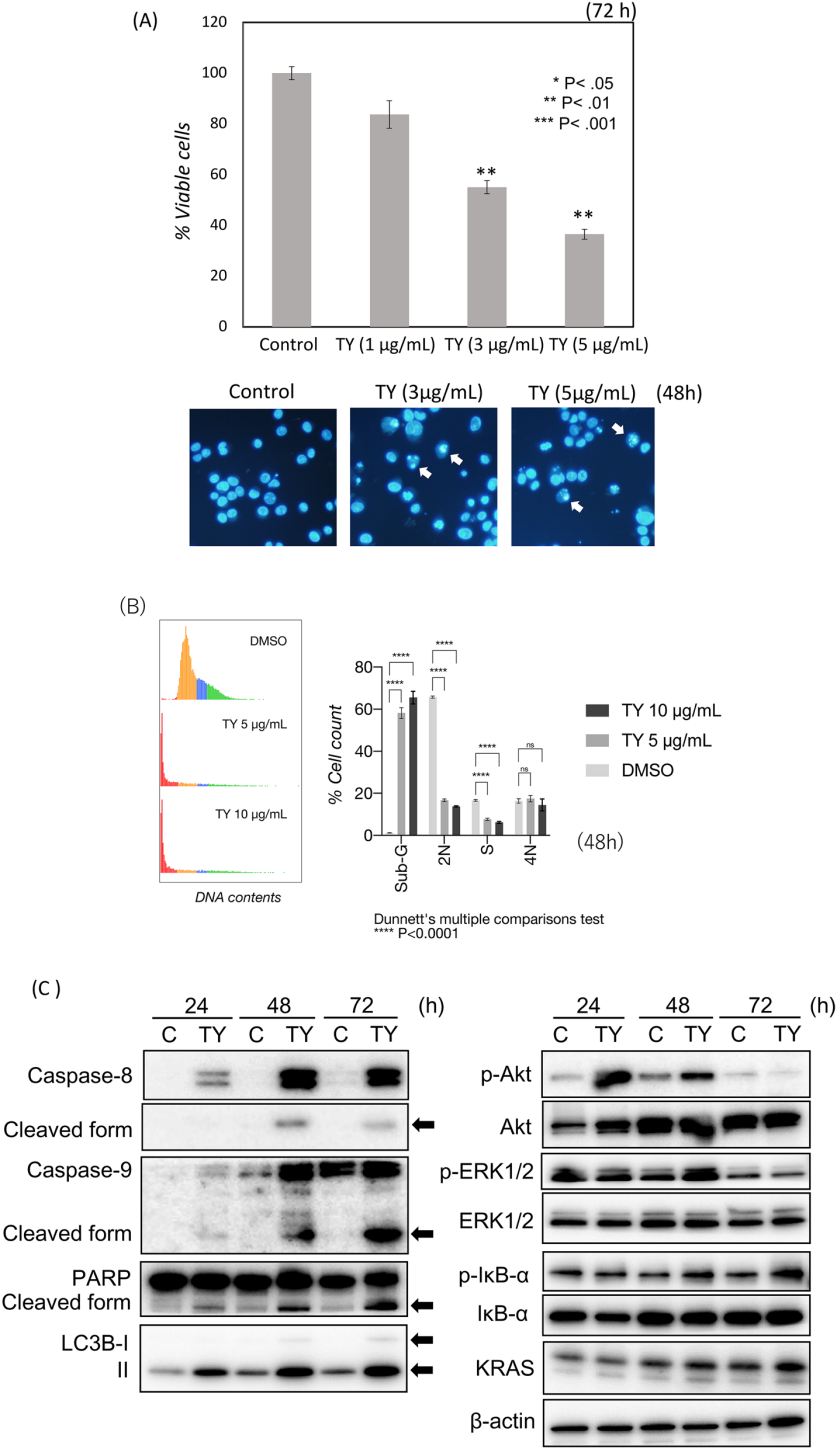

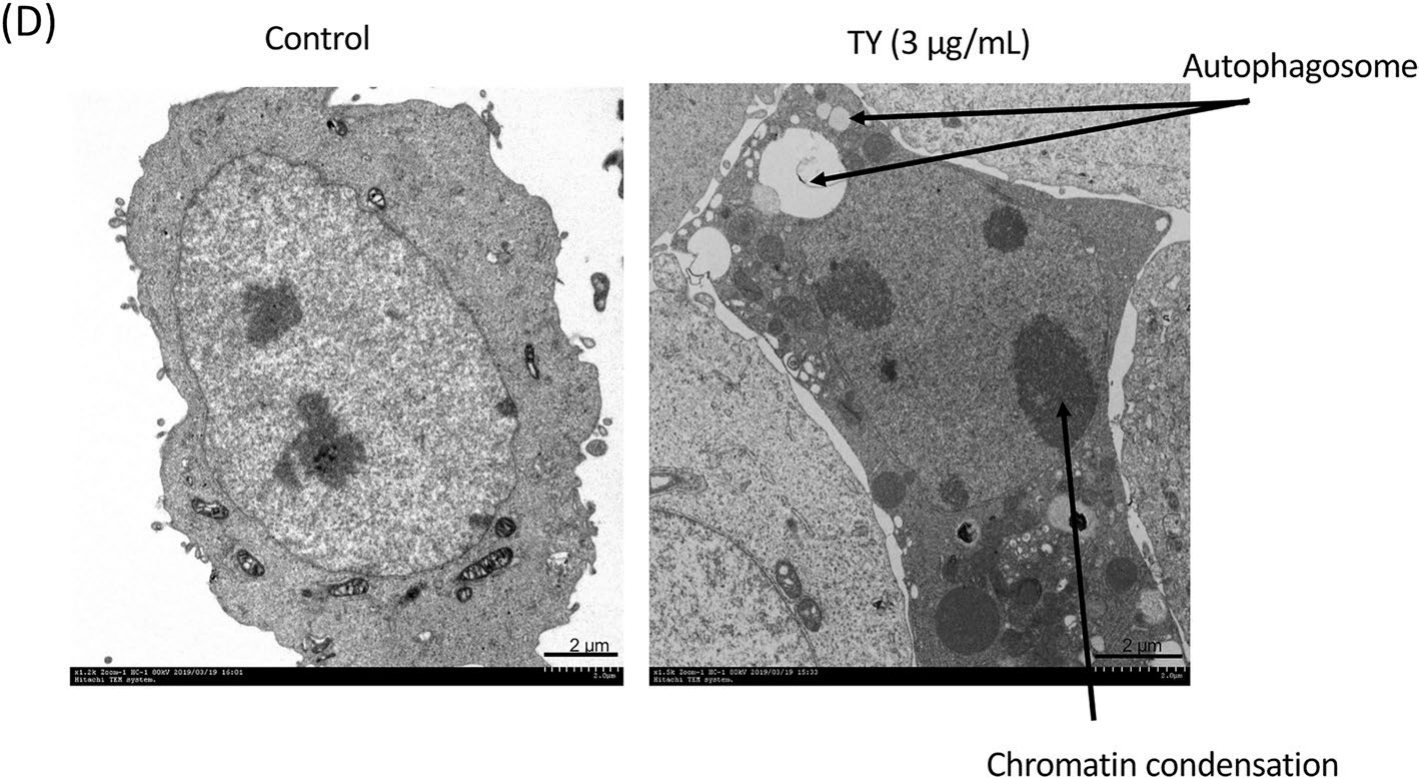
Growth inhibition by TY (the ethanol extract from *Taxus Yunnanensis* wood) in DLD-1 cells. Effects of TY on the cell growth in the colon cancer cell line DLD-1. (**A**) The cell viability was estimated at 72 h after treatment. Data were obtained from at least three independent experiments. Data for the representative concentrations are shown. The cell viability of the control (DMSO alone) is indicated as 100%. The growth inhibitory activity (IC_50_) of TY is indicated in Table 1. *P < .05 is significant. Apoptotic cell death is induced by TY. Apoptotic cells (white arrows) at 48 h after treatment were shown by Hoechst 33,342 staining. (**B**) Cell cycle analysis in TY-treated cells. Cell-count percentage of G0/G1 (2 N), S, and G2/M (4 N) phases for DLD-1 cells treated with TY (5 or 10 µg/mL) or DMSO. ****P < 0.0001, 1-way ANOVA with Dunnett’s post hoc test. Data are presented as the mean ± SD (*n* = 3). NS, not significant. (**C**) Time-dependent activation of the apoptotic signaling cascade in comparison with the growth signal cascade after the treatment with 5 μg/mL of TY, which was estimated by Western blot analysis. Arrows indicates cleaved forms of caspases and LC3B I and II. (**D**) Electron microscopic findings in TY-treated DLD-1 cells. The cells were treated with 3 μg/mL of TY for 72 h.

**Figure 2:**
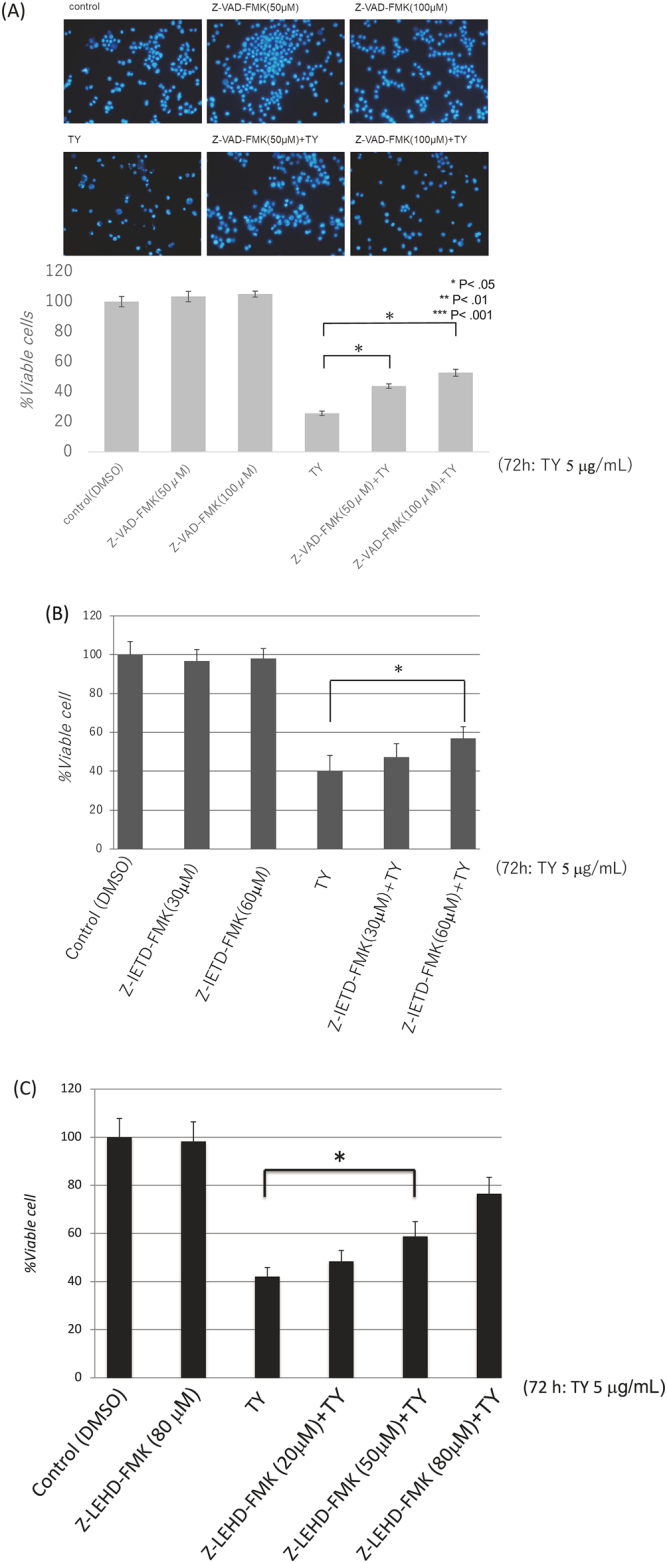
Effects of caspase inhibitors on apoptosis induced by TY (5 μg/mL) in DLD-1 cells. (**A**) Inhibition of TY-induced apoptosis by combined treatment with pan-caspase inhibitor Z-VAD-FMK at 72 h after treatment with TY. Hoechst 33348 staining of treated cells (upper figure). Rescue of the viable cell rate in the TY-treated cells by co-treatment with or without the inhibitor (lower figure). (**B**) Rescue of the viable cell rate in the TY-treated cells by co-treatment with or without caspase-8 inhibitor Z-IETD-FMK at 72 h after the treatment with TY. (**C**) Rescue of the viable cell rate in the TY-treated cells by co-treatment with or without caspase-9 inhibitor Z-LEHD -FMK at 72 h after the treatment with TY.

In order to examine the growth inhibitory effects of the ethanol extract of the wood of *Taxus Yunnanensis* (TY), we tested 9 human cancer cell lines which have mutated *p53* and 4 normal cell lines, as shown in Table 1. The activity was markedly high against the tumor cells; IC50 values ranged from 0.4 to 4 μg/mL, in contrast to the normal cells other than human fibroblast TIG-3–20 cells (Table 1). We also tested 5-fluorouracil (5-FU) and oxaliplatin (OXA)-resistant DLD-1 cells^13^ (Table 1). TY exerted high growth inhibitory activity against the 5-FU or OXA-resistant DLD-1 cells, similar to the original DLD-1 cells (Table 1). Morphologically, all cancer cell lines had apoptotic findings, such as chromatin condensation and nuclear fragmentation, on Hoechst 33342 staining. In order to examine the mechanism of apoptosis, we focused on a human colon cancer DLD-1 cell line among the cell lines because it exhibited typical apoptotic changes by Hoechst 33342 staining (Fig. 1A). Cell-cycle analysis revealed a major population of sub-G1 phase after treatment with 5 μg/mL for 48 h (Fig. 1B), which indicated a typical apoptotic feature. Biochemically, the apoptosis was induced in a caspase-dependent manner, which was evaluated by Western blot analysis (Fig. 1C). The cleaved forms of poly (ADP-ribose) polymerase (PARP) that catalyzed by caspase-3 clearly emerged at 24 h after the treatment, along with the cleaved forms of caspase-8 and caspase-9, suggesting that TY induced apoptosis via both extrinsic and intrinsic pathways. Survival signal pathways of PI3K/Akt and MAPK/Erk were relatively activated up to 48 h after the treatment, but returned to normal at 72 h. The expression levels of KRAS and IκB were also unchanged during 72 h after treatment. A transition from LC3B-1 to LC3B-II, a biochemical marker of autophagy, was observed (Fig. 1C). Electron microscopic study demonstrated typical apoptotic findings accompanied by the formation of several autophagosomes in the same cell (Fig. 1D). Thus, TY induced apoptosis by activating both apoptosis pathways and altering autophagic cell death in DLD-1 cells. Next, we performed cell biological examination using caspase inhibitors. As expected, significant apoptosis inhibitory effects of Z-VAD-FMK, a pan-caspase inhibitor, were noted based on the viable cell rate and Hoechst 33342 nuclear staining (Fig. 2A), similar to the case of the capase-8 inhibitor, Z-IETD-FMK (Fig. 2B). Furthermore, we examined the inhibitory effect of caspase-9 inhibitor, Z-LEHD-FMK on TY-induced apoptosis to verify the signaling via mitochondria. As shown in Fig. 2C, a significant inhibition of the apoptosis was found.

**Table 1:** Antiproliferative activity (IC50 value in μg/mL) of ethanol extract of *Taxus Yunnanensis* wood against proliferating cells.

Cell line	Wood (ethanol extract)	Apoptosis
**Human tumor cell line**		
SH-SY5Y (neuroblastoma)	0.98	+
DU-145 (prostate carcinoma)	1.92	+
PANC-1 (pancreatic carcinoma)	0.81	+
T24 (urinary bladder carcinoma)	2.62	+
MDA-MB-231 (breast adenocarcinoma)	0.56	+
Rh30 (rhabdomyosarcoma)	0.42	+
K562 (myelogenous leukemia)	2.56	+
RPMI8226 (myeloma)	1.2	+
DLD-1 (colorectal carcinoma)	3.81	+
DLD-1/5FU	3.46	+
DLD-1/OXA	3.52	+
**Non-cancerous cell lines**		
ASF-4-1 (human fibroblasts)	20.94	–
H9c2 (rat cardiocytes)	50 <	–
TIG-3-20 (human fibroblasts)	2.89	–
HMEC (human mammary epithelial cells)	50 <	–

**Figure 3:**
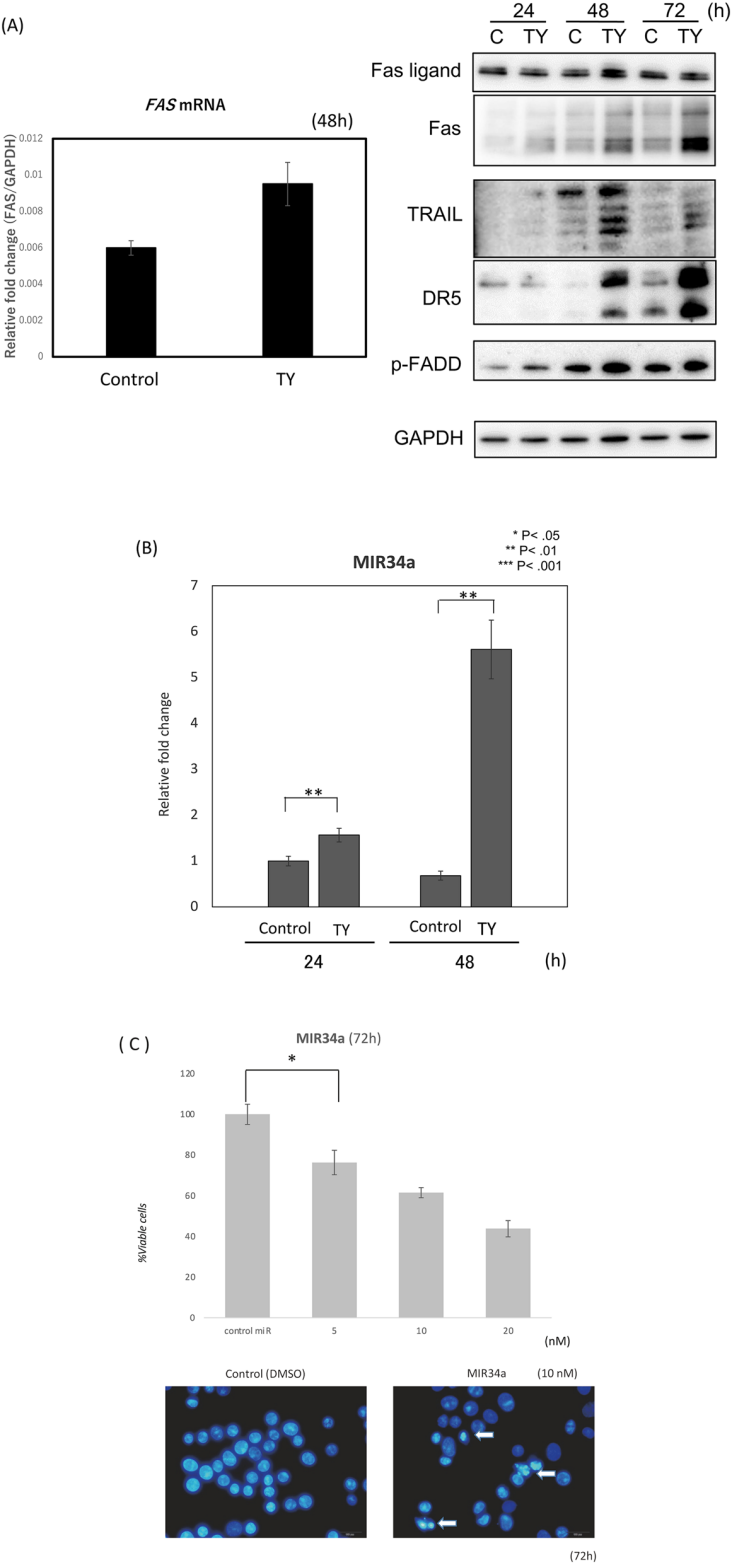

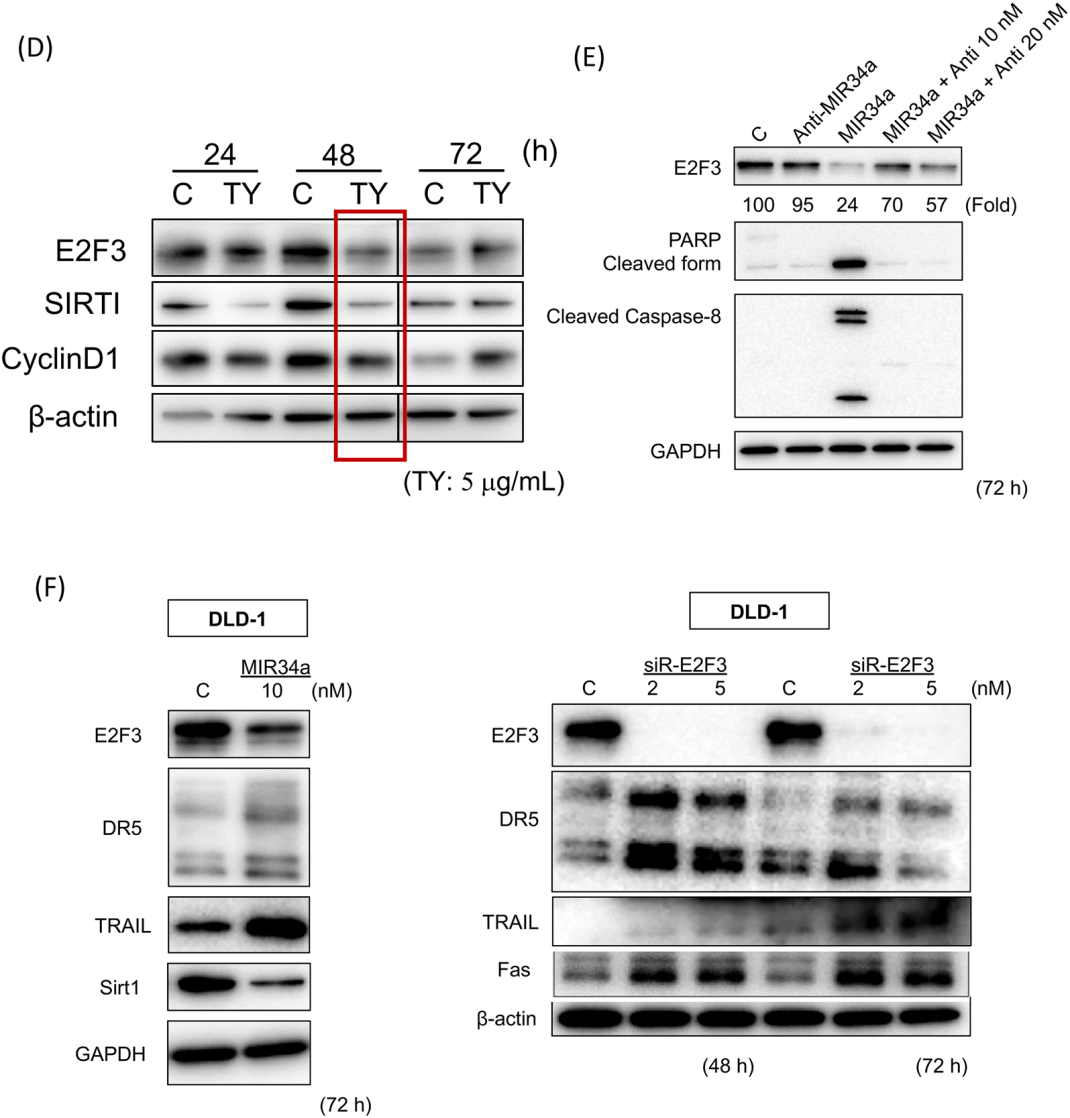
TY (5 μg/mL) induced the expression of the death factors/receptors Fas L/Fas and TRAIL/DR5. (**A**) The expression levels of *FAS* mRNA at 48 h after TY treatment estimated by qRT-PCR (left figure). Time-dependent protein expression of p-FADD and death factors/receptors Fas-L/Fas and TRAIL/DR5 were examined by Western blot analysis. (**B**) Time-dependent expression of MIR34a after treatment with TY estimated by qRT-PCR. Expression levels of MIR34a are shown as the relative ratios with respect to the RNU6B expression levels. (**C**) Ectopic expression of MIR34a inhibited cell growth dose-dependently and induced apoptosis according to Hoechst 33348 staining at 72 h after transfection. The apoptotic cells are indicated by white arrows. (**D**) Expression of MIR34a-targeting E2F3, SIRT1, and CyclinD1 was downregulated by the TY. (**E**) Anti-MIR34a (Anti; 10 and 20 nM) partly canceled the activation of caspase-8 and PARP cleavage elicited by co-transfection with MIR34a. (**F**) Silencing of E2F3 by siR-E2F3 or MIR32a increased in the expression levels of DR5, TRAIL, and Fas.

**Figure 4:**
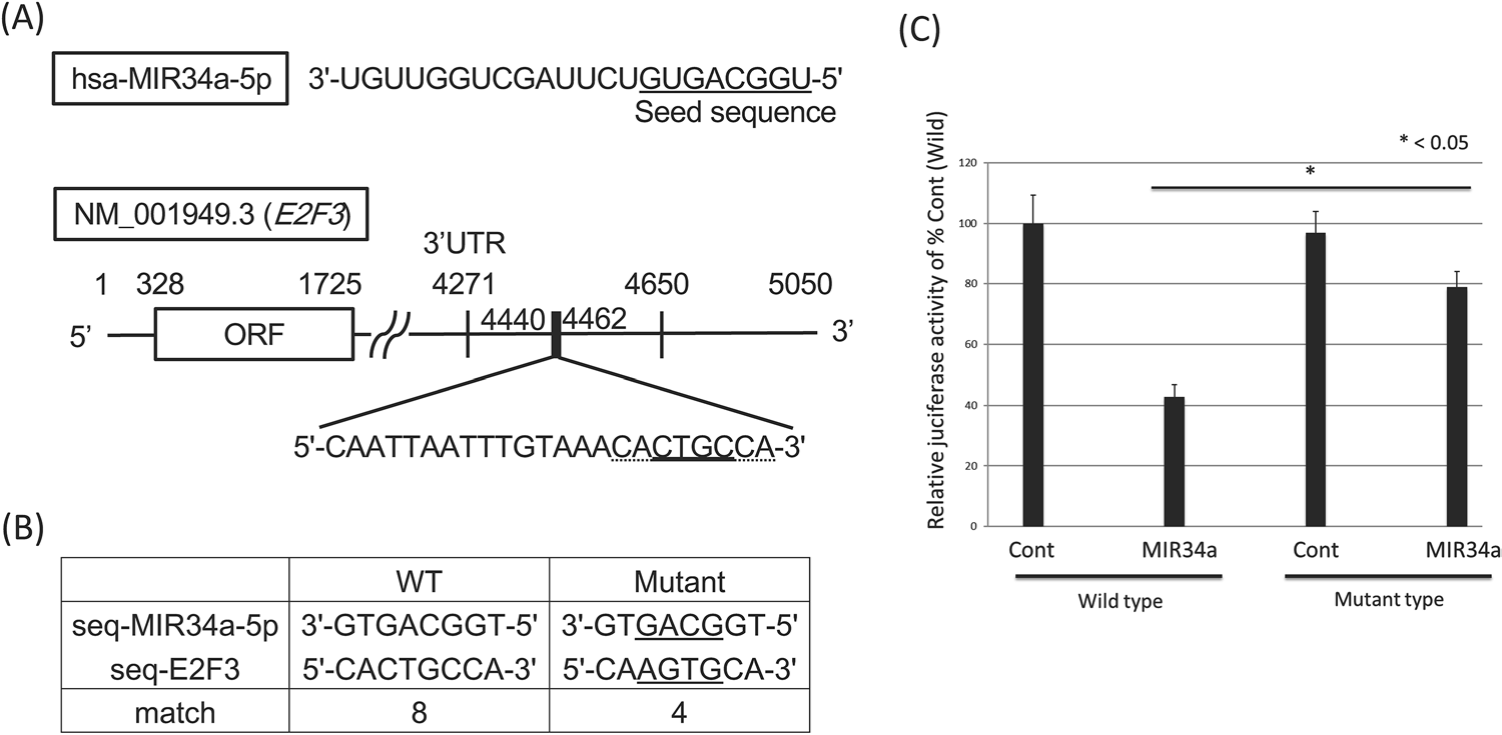
MIR34a binds to *E2F3* mRNA in the Luciferase assay. (**A**–**C**) The ORF region of human *E2F3* mRNA and the predicted binding site for MIR34a (the sequence region 4440–4462) were inserted into a pMIR-REPORT Luciferase miRNA Expression Reporter Vector (wild). The mutant-type pMIR vector contained a mutated seed sequence (from CTGC to AGTG) for MIR34a. Luciferase activities were measured after co-transfection with control RNA or MIR34a (10 nM) and wild or mutant pMIR vector.

**Figure 5:**
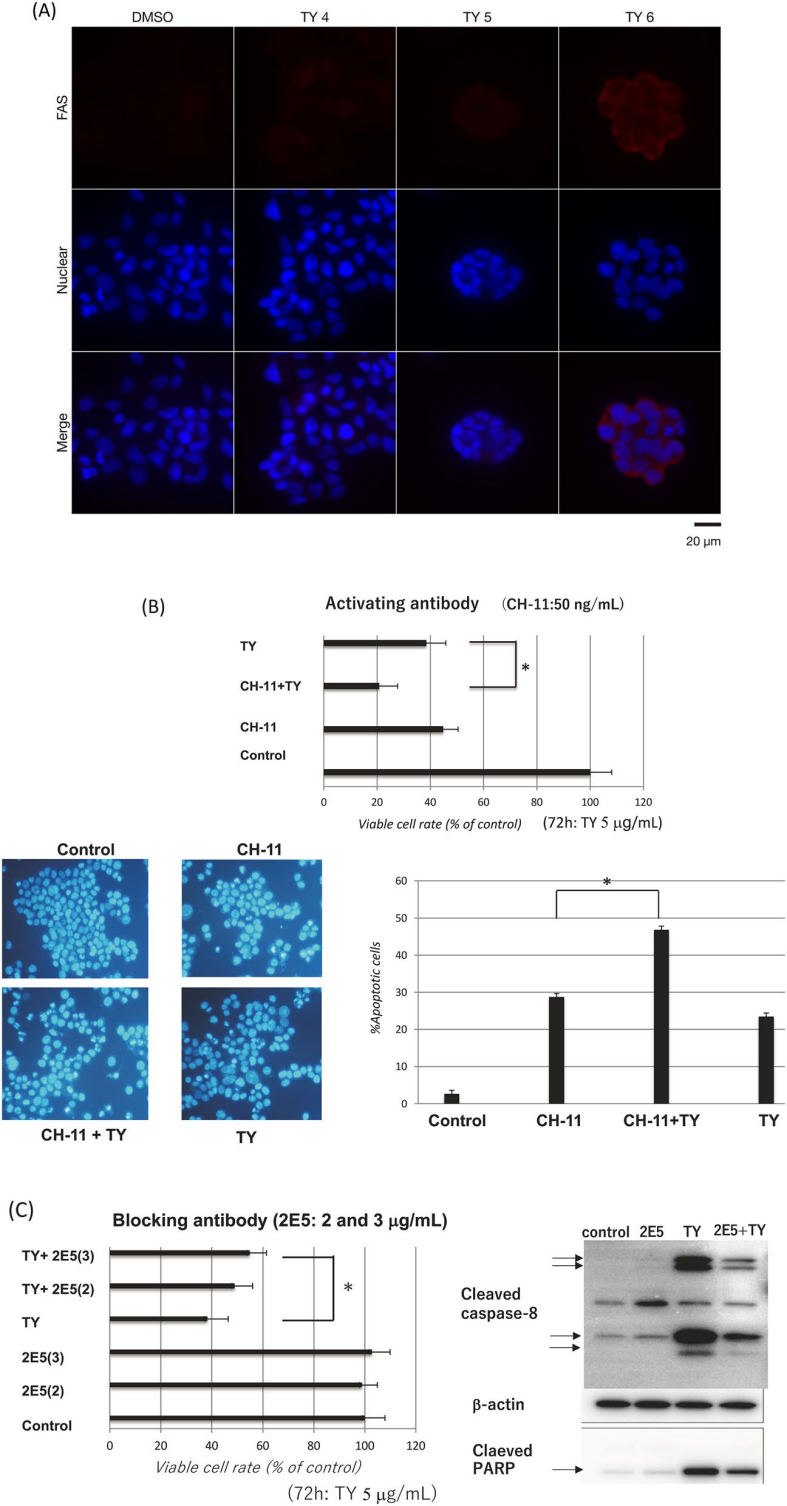
TY upregulated the expression of Fas that functions as a caspase-8-dependent apoptosis inducer. (**A**) Immunocytochemistry for Fas in TY-treated cells. The expression levels of Fas in the cytoplasm increased dose-dependently (4, 5, and 6 μg/mL). Nuclear Hoechst 33348 staining was also performed. (**B**) Fas-activating anti-Fas antibody (CH-11) synergistically induced apoptotic cell death by co-treatment with TY, which was estimated by the viable cell rate and Hoechst 33342 staining. (**C**) TRAIL-blocking anti-TRAIL antibody (2E5) partly canceled apoptotic cell death induced by TY through suppressing the activation of caspase-8, which was estimated by the viable cell rate and Western blot analysis of caspase-8.

Based on the experimental results regarding apoptosis, we next explored which death receptor(s) functioned in the extrinsic apoptosis signaling induced by the wood extract of *Taxus Yunnanensis*. We examined the expression of Fas/FasL and TRAIL/DR5 after treatment. As shown in Fig. 3A, the expression levels of *FAS* mRNA and protein in the treated cells significantly increased at 48 h and the increased protein expression level remained up to 72 h, which may lead to the upregulation of p-FADD (Fig. 3A). Thus, it is confirmed that activation of caspase-8 from death-receptors play a pivotal role in this apoptosis. Of note, the expression levels of TRAIL and DR5 markedly increased at the same time. To examine which transcription factor regulates the expression of FAS, TRAIL, and DR5, we surveyed the changes in the expression levels of certain transcription factors and tumor suppressor miRNAs by Western blot and qRT-PCR, respectively. As a result, we found markedly increased expression levels of MIR34a at 48 h after TY treatment compared with the control by qRT-PCR (Fig. 3B). Accordingly, to assess the role of MIR34a in the growth suppression along with apoptosis, we induced its ectopic expression in DLD-1 cells, which resulted in significant growth inhibition with apoptotic characteristics, such as chromatin condensation and fragmentation, on Hoechst 33342 staining (Fig. 3C). The miRNA database (miRbase) suggested that MIR34a, one of the major tumor suppressor miRNAs, silences *E2F3*, *SIRT1,* and *CyclinD1*. We thus examined the protein expression of the transcription factor E2F3, SIRT1, and CyclinD1 after treatment. The expression of E2F3, SIRT1, and CyclinD1 was significantly downregulated from 24 h up to 48 h (Fig. 3D). Next, the silencing of *E2F3* by MIR32a (20 nM) transfection with the activation of caspase-8 was partly canceled by co-transfection with anti-MIR32a in a dose-dependent manner (Fig. 3E). Thus, the MIR34a/E2F3 axis was established in apoptosis after the treatment with TY. Furthermore, we confirmed the increased expression levels of the death receptors DR5 and TRAIL in the MIR34a-trasfected cells at 72 h (Fig. 3F). We thus examined whether *TRAIL/DR5* are the target genes of E2F3. To assess transcriptional control of *DR5* and *TRAIL* by E2F3, we silenced *E2F3* by siR-E2F3. As expected, DR5 and TRAIL were both upregulated according to Western blot analysis (Fig. 3F). Therefore, E2F3 may control the inhibitory machinery of the expression of *Fas* and *TRAIL/DR5.* The luciferase reporter assay for targeting *E2F3* by MIR34a was performed and the activity was reduced by the ectopic expression of MIR34a, but partly canceled in the case of a mutated binding site (Fig. 4A–C). As for FAS, a dose-dependent increase in the expression level was observed in the cytoplasm by immunocytochemical study (Fig. 5A).

### Functional assays of the increased FAS and TRAIL/DR5 induced by TY using CH-11 and 2E5

**Figure 6:**
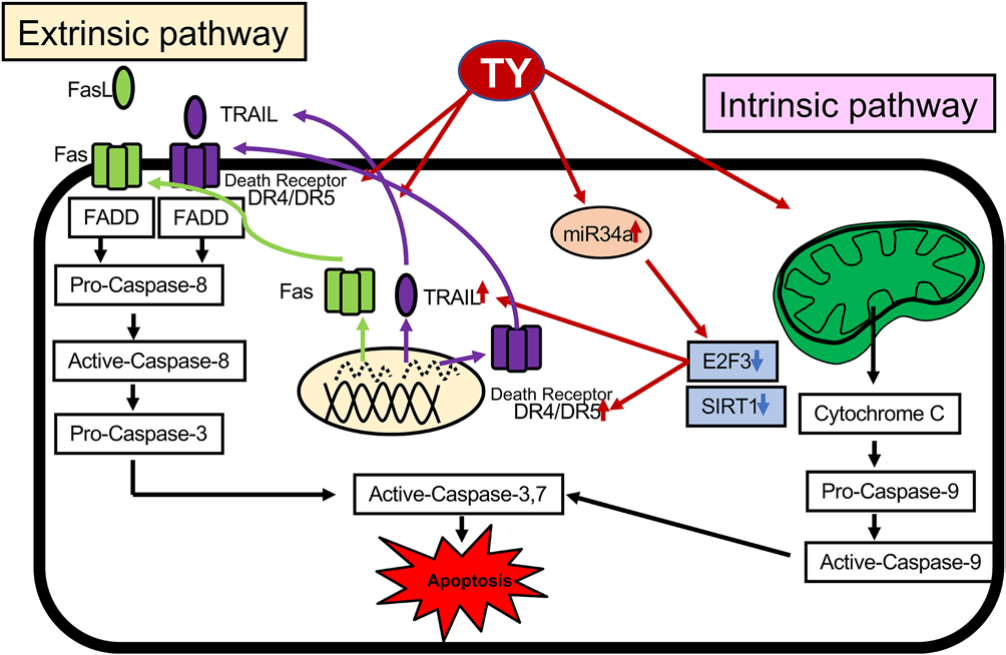
Schematic of the mechanism of TY-induced apoptosis in DLD-1 cells.

We next examined whether the increased expression levels of FAS and TRAIL/DR5 by TY treatment contribute to this apoptosis. Expectedly, the activating antibody for FAS (CH-11)^15,16^ synergistically induced apoptosis based on the viable cell rate and Hoechst 33346 nuclear staining after combined treatment with TY (Fig. 5B). To further confirm whether TY induces TRAIL/DR5-driven apoptosis in the treated DLD-1 cells, we examined whether anti-TRAIL antibody (2E5)^11^ can block the apoptosis elicited by TY. As shown in Fig. 5C, the antibody efficiently blocked the activation of caspase-8 through TRAIL/DR5 binding, similar to the case of the caspase-8 inhibitor. Thus, the upregulated Fas, TRAIL, and DR5 via MIR34a/E2F3 axis function in TY-induced apoptosis (Fig. 6).

### Anti-tumor effects of TY on mouse models

**Figure 7:**
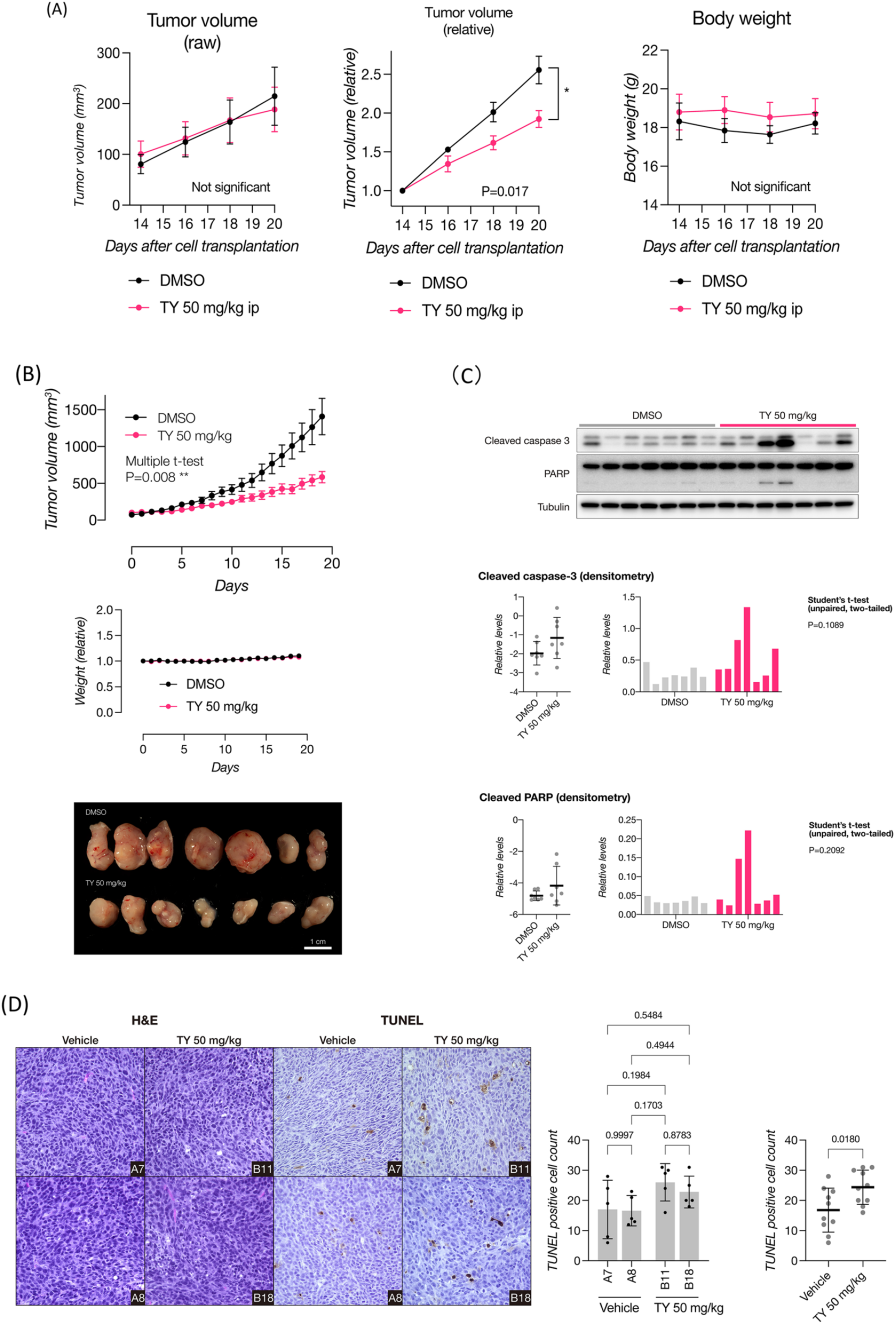
Anti-tumor activity of TY in mouse models. (**A**)Tumor volume (raw and relative) and body weight in the experiments to assess the anti-proliferative effects of TY (50 mg/kg) in the DLD-1 xenograft mouse model (n = 5). Dots indicate the administration of TY. *P < 0.05, two-tailed, unpaired Student’s t-test. Data are presented as the mean ± SEM. (**B**) Tumor volume, body weight, and gross images of tumors to assess the anti-proliferative effects of TY (50 mg/kg) in the Colon 26 syngeneic mouse model (n = 8). **P < 0.01, two-tailed, unpaired Student’s t-test. Data are presented as the mean ± SEM. TY was given by peritoneal injection every day for 20 days. Dots indicate the administration of TY. The tumor samples were analyzed with immunoblots for two apoptosis markers (cleaved caspase 3 and PARP), and the densitometric values are indicated along with the blots. (**C**) Representative images and positive cell count on TUNEL staining of the tumor tissue of mice shown in Fig. 7B. Data are presented as the mean ± SD. *P < 0.05, two-tailed, unpaired Student’s t test.

In order to examine the anti-tumor effects of TY in in vivo mouse models, we produced 2 different mouse models, DLD-1 cell xenograft and syngenic tumor-bearing mice. In DLD-1 cell xenograft mice, the treatment was performed twice a week, for 4 times in total. Significant anti-tumor effects were found in the treated group compared with the control group (Fig. 7A). However, no apoptosis in the syngenic graft tumors of treated mice was observed. In syngenic graft mice, Colon 26 cells were cutaneously inoculated into the back and the extract was intraperitoneally administered every day at a dose of 50 mg/kg. As shown in Fig. 7B, the tumor growth in the treated group was suppressed compared with that in the control (DMSO) group. Protein samples from TY-treated tumors demonstrated the activation of caspase-3 (Fig. 7C). Pathologically, HE staining of the tumor samples confirmed the coexistence of macrophages and apoptotic cells with pyknotic nuclei in the interstitial region. TUNEL study revealed many positive cells in the area (Fig. 7D).

## Discussion

We demonstrated that the natural components included in the wood of *Taxus Yunnanensis* induce apoptotic cell death partly through activating an extrinsic pathway, in which death ligands and receptors were upregulated by TY. TY induced apoptosis in tumor cells in an autocrine or paracrine manner via TRAIL or FasL from treated tumor cells. This suggests that the increased expression levels of Fas and TRAIL/DR5 by TY make tumor cells susceptible to attack by tumor-related immune cells such as cytotoxic T cells and NK cells. We also clarified the mechanism of upregulation of cell death ligands/receptors through the MIR34a/E2F3 axis toward apoptosis. TY markedly increased the expression level of MIR34a, which silenced *E2F3* by 36–48 h after treatment. As expected, decreased expression of E2F3 by siR-E2F3 upregulated Fas and TRAIL/DR5. Thus, E2F3 negatively regulated their mRNA transcription. Furthermore, we noted increased expression of Fas in the cell cytoplasm by immunocytochemistry 48 h after the treatment with TY (Fig. 5A), and anti-Fas activating antibody (CH-11) synergistically promoted the growth inhibitory activity when combined with TY (Fig. 5B). On the other hand, the antibody against TRAIL efficiently inhibited the apoptosis elicited by TY, which suggested the release of TRAIL from the DLD-1 cells treated with TY (Fig. 5C). The MIR34a/E2F3/death ligand-receptor cascade induced by TY functions in the apoptosis. Thus, TY induced apoptosis through MIR34a/E2F3, leading to death ligand-receptor cascades.

miRNAs are a group of small non-coding RNAs functioning in target gene regulation at the transcriptional or post-transcriptional level. Accordingly, miRNA can function as an oncogene or tumor suppressor by regulating growth or death-associated genes in human cancer cells. MIR34a is a typical tumor suppressor miRNA, whose transcription factor is p53, which is deleted in most cancers and DLD-1 cells^14^. The current study suggested that these bi-directional circuits exist widely among miRNA/target transcription factor genes^15–17^. Reinforcing the inhibition of transcripts in specific target cells is one prominent biological function of miRNAs. Some miRNAs silence *E2F3* and several of them are also regulated by E2F3 through an auto-regulatory feedback loop^18^. We revealed that MIR34a targets E2F3 by Western blot analysis and Luciferase assay. As the ectopic expression of MIR34a and siRNA silencing of *E2F3* both upregulated Fas and TRAIL/DR5 protein expression, miR34a/E2F3/Fas and TRAIL/DR5 cascades may exist. Phytochemicals impact epigenetic modifications, such as DNA methylation, and histone modifications, in addition to the regulation of non-coding miRNA expression for the prevention of cancer^7,8^. Due to their significant roles in cell physiology, expression level alterations are directly related to cancer progression. In this study, the phytochemicals of TY increased the expression level of MIR34a, which may be associated with demethylation of the promoter region of MIR34a because the promoter of MIR34a is frequently hypermethylated in colon cancer cells^19–21^. Many phytochemicals have promise in altering DNA methylation and histone modifications in carcinogenesis, which may lead to the use of dietary-based phytochemicals as potent and effective chemopreventive medicines.

TY induced the autocrine and/or paracrine release of FasL/Fas and TRAIL/DR5 in treated DLD-1 cells. As for TRAIL signaling, TRAIL-R2/DR5 was used for apoptosis in this case because the TRAIL antibody blocked the activation of caspase-8. The induction of apoptosis can be classified into either intrinsic apoptosis, triggered by p53 in response to cellular damages^22^, or extrinsic apoptosis induced upon death ligand binding to a death receptor. Conventional anticancer drugs induce intrinsic apoptosis through p53 in response to cellular damage^22,23^. However, most tumors have mutations in p53, leading to its inactivation and failure of chemotherapeutic agents. In contrast, TRAIL-induced apoptosis remains possible in many tumors despite non-functional p53. Therefore, the effects of TY on TRAIL signaling and sensitization may be useful for cancer prevention and therapy. Two extrinsic pathways from death-receptors can be distinguished based on the requirement of cross-signaling mitochondrial apoptotic machinery^24^. In type I cells, DISC activation is sufficient to effectively induce the full caspase cascade. In type II cells, DISC activation causes caspases-9 and -3 activation by signaling pathways via mitochondria. In this case, the mitochondria membrane potential is not downregulated and activation of caspase-9 is clearly observed. In addition, caspase-9 inhibitor considerably blocked the apoptosis. It is reasonable that the extrinsic pathway of type II is the major machinery in this apoptosis. When tumor necrosis factor (TNF)-related apoptosis-inducing ligand (TRAIL) was discovered in 1995, many studies were performed to demonstrate that it selectively induces apoptosis in tumor cells in vivo^25–27^. At present, TRAIL-related therapy against human cancers is under investigation. The use of TY with the ability to selectively kill tumor cells through the TRAIL-related signaling pathway is preferable. On the other hand, as shown in Fig. 1C, the expression levels of Akt/p-Akt, ERKs/p-ERKs and KRAS at 72 h after the TY treatment almost unchanged. Therefore, the survival signals from growth factors such as epidermal growth factor (EGF) and fibroblast growth factor (FGF) would sustain during activation of the extrinsic pathway. Thus, it seemed that TY does not affect the survival signal pathways. Thus, our study suggested that the wood extract from TY selectively kills tumor cells by inducing apoptosis through the increased expression of cell-death ligands/receptors, which also make the marked tumor cells susceptible to the immunosurveillance system. The similar findings were also observed in other colon cancer cell lines.

TY contains many diterpenoids, such as baccatin III, 10-deacetyltaxuyunnanine C, and taxuyunnanine E, which may have anti-proliferative effects^5^. However, most are cytotoxic to healthy cells. Cytotoxic 10-deacetyltaxuyunnanine C induced the activation of caspase-8, but not the increased expression of cell-death factor/receptors. The anti-cancer effects of TY may be due to synergy between diterpenoids and other factors such as lignans.
